# Electrochemical Investigations of Double Perovskite M_2_NiMnO_6_ (Where M = Eu, Gd, Tb) for High-Performance Oxygen Evolution Reaction

**DOI:** 10.3390/nano13233076

**Published:** 2023-12-04

**Authors:** Kiran P. Shinde, Harish S. Chavan, Amol S. Salunke, Jeongseok Oh, Abu Talha Aqueel Ahmed, Nabeen K. Shrestha, Hyunsik Im, Joonsik Park, Akbar I. Inamdar

**Affiliations:** 1 Department of Materials Science and Engineering, Hanbat National University, Daejeon 34158, Republic of Korea; 2 Department of Chemistry and Chemical Engineering, Inha University, 100 Inha-ro, Michuhol-gu, Incheon 22212, Republic of Korea; 3 Division of Physics and Semiconductor Science, Dongguk University, Seoul 04620, Republic of Korea

**Keywords:** electrocatalysis, water splitting, oxygen evolution reaction, double perovskite, electrochemical properties

## Abstract

Double perovskites are known for their special structures which can be utilized as catalyst electrode materials for electrochemical water splitting to generate carbon-neutral hydrogen energy. In this work, we prepared lanthanide series metal-doped double perovskites at the M site such as M_2_NiMnO_6_ (where M = Eu, Gd, Tb) using the solid-state reaction method, and they were investigated for an oxygen evolution reaction (OER) study in an alkaline medium. It is revealed that the catalyst with a configuration of Tb_2_NiMnO_6_ has outstanding OER properties such as a low overpotential of 288 mV to achieve a current density of 10 mAcm^−2^, a lower Tafel slope of 38.76 mVdec^−1^, and a long cycling stability over 100 h of continuous operation. A-site doping causes an alteration in the oxidation or valence states of the NiMn cations, their porosity, and the oxygen vacancies. This is evidenced in terms of the Mn^4+^/Mn^3+^ ratio modifying electronic properties and the surface which facilitates the OER properties of the catalyst. This is discussed using electrochemical impedance spectroscopy (EIS) and electrochemical surface area (ECSA) of the catalysts. The proposed work is promising for the synthesis and utilization of future catalyst electrodes for high-performance electrochemical water splitting.

## 1. Introduction

The inadequate reserves of non-renewable fossil fuels such as oil, coal, and gas, which have created environmental problems, have forced us to search for alternative energy sources that could be extracted from renewable natural resources like water, the sun, and wind. Hydrogen is considered to be an alternative energy source to fossil fuels because of its excellent properties such as carbon neutrality during the combustion process, high energy density, sustainability, high efficiency, and environmental friendliness [1,2,3,4]. It can be produced in many different ways, such as natural gas reforming (thermal process), steam-methane reforming, biomass mass and coal gasification, and electrochemical water splitting. Among these, electrocatalysis (electrochemical water splitting) is one of the cleanest and most inexpensive ways to produce hydrogen from abundantly available water [5,6,7,8]. It is a simple process of splitting water into molecular oxygen and hydrogen using catalytic electrodes via an electrolysis process. It consists of an anode and cathode, where oxygen evolution reactions (OERs) and hydrogen evolution reactions (HERs) take place, respectively. Among these processes, an OER is considered to be a bottleneck in water splitting because of its sluggish kinetics, and the requirement of a high overpotential needs to be resolved. To overcome this issue, it has become very important to develop a catalytic electrode material with low overpotentials and faster reaction kinetics. Several precious metals like platinum (Pt), iridium oxide (IrO_2_), and ruthenium oxide (RuO_2_) have been studied, but they suffer the problems of high cost and relative scarcity, limiting their widespread use [9]. Moreover, many other metal oxides, sulfides, phosphides, and their complexes based on Ni, Fe, Mo, Co, Cu, and Mn have been investigated for efficient water-splitting activity [7,8,9,10,11,12,13,14,15]. However, there are still many challenging issues to overcome like high overpotential, complicated synthesis processes, low current density, and electrochemical stability in acidic and alkaline media.

Double perovskites with the general formula A_2_B_1_B_2_O_6_, in which A_2_ is lanthanide or alkali earth metals, B_1_ and B_2_ are transition metals positioned at the center of the octahedron with six coordination oxygen ions, have outstanding chemical and physical properties [16]. It is very important to note that A_2_B_1_B_2_O_6_ possesses more abundant combinations because of different electron configurations, flexible band structure, favorable redox behavior, and different ion radii of B_1_ and B_2_, making it favorable for electromagnetism and catalytic properties [17,18,19]. Various synthesis routes such as the facile hydrothermal/solvothermal route, solid-state reaction, and wet chemical sol-gel process have been employed to fabricate double perovskite oxides [16,20,21,22]. It has been reported that double perovskite oxides have better catalytic properties than single perovskite oxides due to the high lattice oxygen, excellent reproducibility, and synergistic effect between transition metals. Additionally, it embraces a periodic structure compared with the doped perovskite oxides, which helps to protect the lattice distortion and improve cycling stability. Thus, the unique structure of the double perovskite oxides having octahedron unit cells like B_1_O_6_ and B_2_O_6_ results in a large number of active sites and a favorable electronic structure, facilitating the charge transfer process during the electrocatalysis process [23,24]. Additionally, some inorganic lead-free Mn-based double perovskites have also been investigated for photovoltaic applications and oxygen storage technology [25,26]. This suggests the multifunctionality of the Mn-based double perovskites in coping with the growing interest in environmentally friendly earth-abundant materials for mass production. Nevertheless, there is still scope to improve the catalytic properties of the double perovskite oxides by tuning the composition and doping with some other elements in B_1_ and B_2_ sites for the acceleration of ion transport. So far, double perovskite oxides based on M_2_NiMnO_6_ (where M = Eu, Gd, Tb) have not yet been clearly investigated for electrochemical water-splitting properties. Moreover, the A-site doping does not affect the electronic structure, but it can alter the oxidation or valence states of the NiMn cations, porosity, and oxygen vacancies.

In this work, we fabricate double perovskite oxides based on M_2_NiMnO_6_ (where M = Eu, Gd, Tb) via solid-state reaction methods and they are used to study water-splitting properties in terms of the oxygen evolution reaction. The formation of the different double perovskite structures is well supported by the XRD, XPS, and Raman analyses. It is found that Tb_2_NiMnO_6_ has outstanding OER properties, exhibiting a low overpotential (288 mV at 10 mAcm^−2^) and Tafel slope (38.76 mVdec^−1^). All the catalytic electrodes have excellent electrochemical stability at different current rates for more than 100 h in an alkaline medium, suggesting their widespread use in commercial electrolyzers.

## 2. Experimental Section

### 2.1. Preparation of the Double Perovskites and Their Characterization

Lanthanide series metal-doped double perovskite M_2_NiMnO_6_ (M = Eu, Gd, Tb) compounds were synthesized via the conventional solid-state reaction method. The precursor materials Europium oxide (Eu_2_O_3_), Gadolinium oxide (Gd_2_O_3_), Terbium oxide (Tb_2_O_3_), Nickel oxide (NiO), and Manganese oxide (MnO_2_) (Sigma Aldrich, St. Louis, MI, United States, Purity > 99%) were taken in proper stoichiometric proportions. The mixture of the above-mentioned precursors was grounded rigorously using an agate mortar and pestle followed by calcination at 1100 °C for 24 h. The powders were reground and finally sintered at 1300 °C for 48 h. The phase purity and crystallographic structure of the compounds were determined by XRD patterns with CuKα radiation (1.5406 Å) and analyzed by Rietveld refinement using TOPAS software. The morphology and composition were investigated by Scanning Electron Microscopy (SEM, model No S-4700, made by Hitachi, Hitachi City, Japan) and X-ray photoelectron spectroscopy (XPS, VersaProbe, PHI 5000). The Raman measurements of the double perovskite compounds were carried out at room temperature over the wavenumber range of 100–1500 cm^−1^ using a Horiba LabRAM HR-(Kyoto, Japan) 800 spectrometer.

### 2.2. Fabrication of the Catalytic Electrodes and Electrochemical Measurements

The catalytic electrodes were fabricated using the doctor blade technique using slurry made with the double perovskite materials. Polyvinylidene fluoride (PVDF) was used as a binder and N-Methyl-2-pyrrolidone (NMP) as a solvent in proper proportions. All the perovskite films were uniformly coated onto stainless steel substrates with an area of 1 cm^2^. The prepared electrodes were dried at 60 °C overnight to evaporate NMP from the electrodes and they were further used for electrocatalysis measurements. The electrocatalysis measurements were carried out using a three-electrode system with electrodes prepared with perovskite materials as working electrodes, Pt wire as a counter electrode, and a saturated calomel electrode as a reference electrode. Electrochemical techniques such as cyclic voltammetry (CV), linear sweep voltammetry (LSV), and chroamperometry (CA) were used to study the electrocatalysis of the prepared materials in 1 M KOH electrolyte. All the LSV curves were measured at a scan rate of 5 mVs^−1^ at room temperature. The electrochemical stability was obtained using CA at a fixed current density (j) over 100 h. The electrochemical surface area (ECSA) of the electrodes was estimated from the CV curves measured at different scan rates of 10, 20, 30, 40, 50, 60, 70, 80 90, and 100 mVs^−1^. All the measured potentials were further converted to a reversible hydrogen electrode (RHE) using the standard conversion formula.

## 3. Results and Discussion

**Figure 1:**
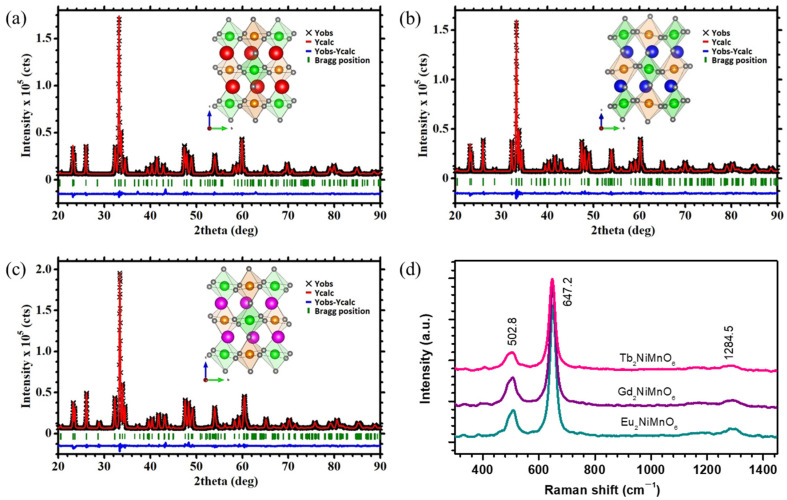
Rietveld refined X-ray diffraction patterns of the double perovskite M_2_NiMnO_6_ (M = Eu, Gd, Tb), (**a**) Eu_2_NiMnO_6_, (**b**) Gd_2_NiMnO_6_, (**c**) Tb_2_NiMnO_6_; inset of each figure defines atomic arrangements in the corresponding monoclinic structures. (**d**) Overlapping of the Raman spectra of all three double perovskite catalysts.

The structural properties of the double perovskite M_2_NiMnO_6_ (M = Eu, Gd, Tb) are investigated via X-ray diffraction (XRD) and Raman spectroscopy measurements. Figure 1a–c depict the Rietveld-refined XRD patterns of the M_2_NiMnO_6_ (M = Eu, Gd, Tb). The diffraction peaks that appeared in the XRD patterns revealed that all the samples have a monoclinic crystal system with space group P2_1_/n [27]. Rietveld refinement suggests that with an increase in the atomic number (Eu (63)→Gd (64)→Tb (65)), crystal density increases (7.65→7.86→7.98 g/cm^3^) and cell volume decreases (222.9→221.6→219.6 Å^3^). We provided the structural parameters determined after the Rietveld refinement of the XRD patterns for M_2_NiMnO_6_ (M = Eu, Gd, Tb) in Table S1 (Supporting Information), portraying the correlation of cell parameters/volume decreases with the change in the ionic radius of M = Eu, Gd, Tb. The insets of Figure 1a–c represent the atomic arrangements in the corresponding monoclinic structures. The detailed analysis of the structural properties of the double perovskites M_2_NiMnO_6_ (M = Eu, Gd, Tb) is discussed in our previous report [16]. Moreover, the structural stability of the double perovskite was determined by estimation of the Goldschmidt tolerance factor (*t*), which was found to be less than 1 for all the samples, suggesting outstanding stability of the samples. Moreover, the structural analysis was also carried out using Raman spectra of all the double perovskites, shown in Figure 1d. The observed band patterns are the typical characteristics of partially disordered double perovskite structures [28] The Raman bands observed at the wavelengths of 502.8, 647.2, and 1284.5 cm^−1^ are associated with the anti-stretching/bending motions, stretching vibrations, and combination and overtone modes of the fundamental, respectively [28,29]. This suggests the formation of phase-pure double perovskite structures.

**Figure 2:**
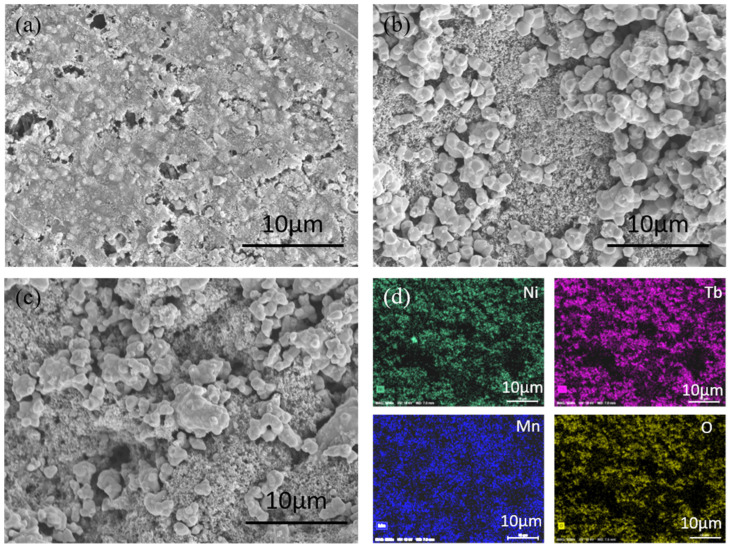
Scanning Electron Microscopic images of the double perovskite M_2_NiMnO_6_ (M = Eu, Gd, Tb), (**a**) Eu_2_NiMnO_6_, (**b**) Gd_2_NiMnO_6_, (**c**) Tb_2_NiMnO_6_, and (**d**) elemental mapping images of the Tb_2_NiMnO_6_ obtained from the EDAX analysis in which the uniform distribution of all the constituent elements such as Ni (green), Mn (blue), Tb (pink), and O (yellow) is observed.

Surface morphology is one of the important factors for the electrocatalysis process which is correlated with the electrochemical surface area (ECSA) of the electrode. It is noted that the nanostructure morphology can provide a larger surface, facilitating better electrochemical properties. Figure 2a–c show the scanning electron microscopic (SEM) images of the slurry-coated double perovskite M_2_NiMnO_6_ (M = Eu, Gd, Tb) catalyst. Eu_2_NiMnO_6_ depicts (Figure 2a) compact surface morphology with clusters and pores. Gd_2_NiMnO_6_ (Figure 2b) and Tb_2_NiMnO_6_ (Figure 2c) have similar surface morphologies of the mixed granular structures with different sizes, which are found to be agglomerated in the form of clusters. Figure 2d shows the elemental mapping images of the representative Tb_2_NiMnO_6_ sample obtained from the energy-dispersive X-ray (EDAX) analysis. It showed the uniform distribution of all the constituent elements such as Ni (green), Mn (blue), Tb (pink), and O (yellow) in the sample. Corresponding EDAX spectra and collective elemental mapping images are shown in Figure S1a,b (Supporting Information).

**Figure 3:**
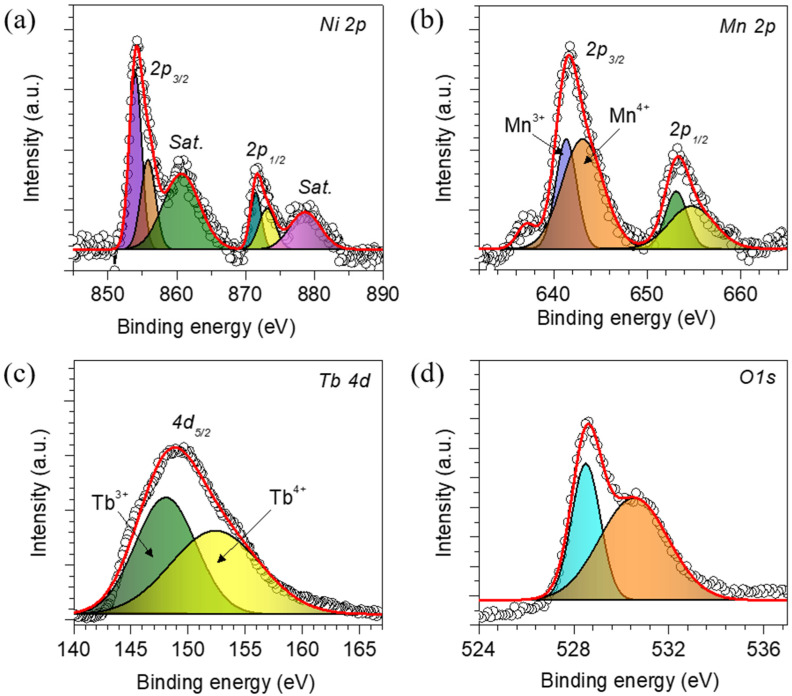
X-ray photoelectron spectroscopy (XPS) curves of the best-performing double perovskite Tb_2_NiMnO_6_ catalyst electrode to determine the surface oxidation states. Deconvoluted core-level XPS spectra of the (**a**) Ni 2p, (**b**) Mn 2p, (**c**) Tb 4d, and (**d**) O 1s.

Surface chemical oxidation states of the double perovskite Tb_2_NiMnO_6_ are estimated using X-ray photoelectron spectroscopy (XPS) analysis. It is noted that the oxidation states of the catalytic electrode at the surface are very important when undergoing the actual catalysis process and surface reconstruction. Figure 3a–d show the XPS spectra of the double perovskite Tb_2_NiMnO_6_, whereas its survey spectra are shown in Figure S2 (Supporting Information). The survey spectra verified all the expected elements in the catalyst. Figure 3a shows deconvoluted core-level Ni 2p XPS spectra, which are further fitted into six peaks. Ni2p_3/2_ possesses two peaks at 854.0 and 855.7 eV associated with the Ni^+2^ and Ni^3+^ oxidation states along with one satellite peak, respectively. Similarly, Ni2p_1/2_ also has peaks of Ni^+2^ and Ni^3+^ at 871.4 and 873.2 eV, respectively, with a satellite peak at 878.5 eV [30]. The XPS spectra of the Mn 2p (Figure 3b) can be fitted into four peaks at 641.4 and 653.1, and also 643.2 and 654.8 eV, which are assigned to Mn^3+^ and Mn^4+^, respectively [31]. From the previous study, it has been noted that the surface oxidation states are distinctly induced by the A-site rare-earth element, suggesting the superexchange mechanism between Ni^2+^ and Mn^4+^. Thus, the ratio of Mn^4+^/Mn^3+^ could be an effective factor in the OER property of the catalysts. This uncertainty in structural stability depending on the A-site element may influence the electrochemical properties of the catalysts. The deconvoluted XPS Tb 4d spectra (Figure 3c) exhibit two peaks at 147.9 and 152.4 eV, which are related to the existence of the Tb^3+^ and Tb^4+^ oxidation states, respectively [32]. Moreover, the O 1s spectra, shown in Figure 3d, have two peaks at 528.7 and 530.6 eV, indicating the exitance of two different oxygen species in the catalyst [33].

**Figure 4:**
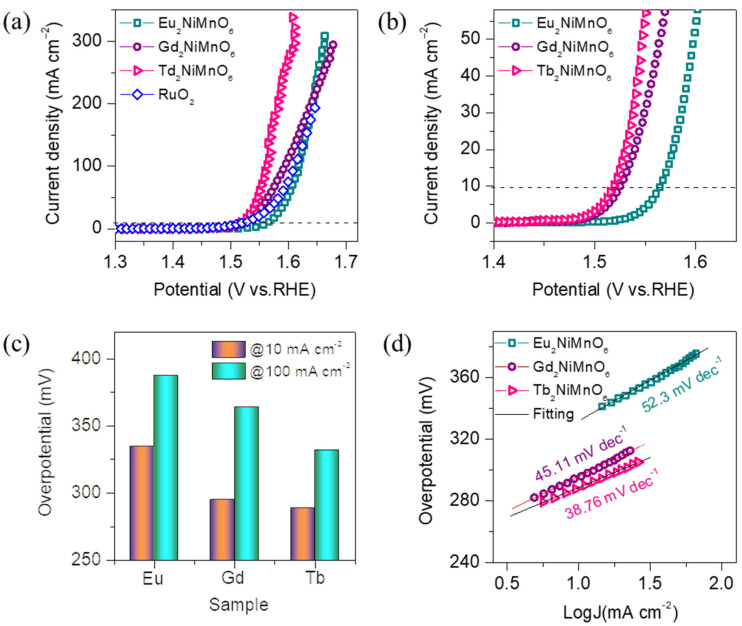
Electrochemical OER properties of the double perovskite M_2_NiMnO_6_ (M = Eu, Gd, Tb), catalysts measured in 1M KOH electrolyte. (**a**) *iR*-corrected OER polarization curves recorded at a scan rate of 5 mVs^−1^, along with the LSV for a benchmark RuO_2_/NF catalyst. Its enlarged view is shown in (**b**), (**c**) overpotentials required to reach a current density of 10 and 100 mAcm^−2^, (**d**) Tafel slopes for the OER.

We investigated the oxygen evolution reaction (OER) properties of double perovskite M_2_NiMnO_6_ (M = Eu, Gd, Tb) catalyst electrodes in 1 M KOH electrolyte. Prior to the OER test, the catalyst electrodes were activated using the cyclic voltammetry (CV) technique for more than 50 cycles. Once the catalyst electrodes were activated, they were subjected to OER measurements using linear sweep volumetry (LSV) at a scan rate of 5 mVs^−1^. All the LSV curves presented in this work are 90 % *iR*-corrected. Figure 4a shows LSV curves of the M_2_NiMnO_6_ (M = Eu, Gd, Tb) catalyst electrodes, whereas Figure 4b depicts its enlarged view. For comparison purposes, an LSV curve of the commercial RuO_2_ catalyst was also included along with the studied perovskite catalysts. A systematic change in the LSV curves with the different A-site rare-earth element doping was detected. It was observed that the Tb_2_NiMnO_6_ exhibited enhanced OER activity compared with that of the other two catalysts. The estimated overpotentials of all the catalyst electrodes are reported in Table S2 (Supporting Information). The overpotential of Tb_2_NiMnO_6_ was found to be 288 mV, whereas for the Eu_2_NiMnO_6_ and Gd_2_NiMnO_6_, it was 334 and 292 mV, respectively, at a current density of 10 mA cm^−1^. As we have discussed in the XPS analysis and also reported in the literature, the enhanced OER properties could be due to the alteration of the oxidation or valence states of the NiMn cations upon A-site rare-earth element doping with different ionic radii [34,35]. The OER overpotential observed for the Tb_2_NiMnO_6_ catalyst was also superior to that of previously reported catalysts in 1M KOH and NaOH electrolytes including, PrBaCo_2_O_6-δ_ (360 mV) [36], La_2-x_SrxNiMnO_6_ (x = 0.6) (367 mV) [37], NdBaMn_2_O_5.5_ (430 mV) [38], Ba_1-x_Gd_1-y_La_x+y_Co_2_O_6+δ_ (470 mV) [39], and BaGdCo_1.8_Fe_0.2_O_6_ (477mV) [40].

The overpotentials at different current densities of 10 and 100 mA cm^−1^ are presented in the histograms shown in Figure 4c. It was noted that the trend of OER properties at higher current densities was similar to that of the lower current density with respect to the A-site doping. Moreover, the lowest overpotential at higher current densities could be beneficial to fabricate and compete with industrial-grade electrolyzers. It has been noted from the literature that the modulated electronic states and surface electronic oxidation states of the cations are important in enhancing the OER activity of the catalysts [41]. Thus, the modulated electronic states in the form of oxygen vacancies could enhance the water adsorption and dissociation kinetics of the intermediates and the active metal sites [42]. In this study, it was observed that the oxidation states of the Mn cation were modified due to the A-site doping with various lanthanide series metals. This was determined in terms of the Mn^4+^/Mn^3+^ ratio. The estimated ratio of Mn^4+^/Mn^3+^ was found to be 1.02, 0.76, and 1.64 for Eu_2_NiMnO_6_, Gd_2_NiMnO_6_, and Tb_2_NiMnO_6_, respectively. Similar results have been presented in our previous report, which studied the enhancement of the magnetocaloric properties of various double perovskites upon lanthanide metal doping. The results suggest that the Mn^4+^ oxidation state is dominant in the best-performing Tb_2_NiMnO_6_ catalysts compared with that of the other two catalysts. Thus, the change in the oxidation states induced by A-site doping of Tb metal indicates the superexchange mechanism of the Ni^2+^–O–Mn^3+^ and Ni^2+^–O–Mn^4+^. This superexchange mechanism of the change in the oxidation state of the metal cations facilitates the OER reaction kinetics, revealing a strong positive correlation between the higher oxidation state of the metal ions and OER electrocatalysis. Hence, the alteration or optimization of the surface oxidation states is an effective strategy to develop efficient electrocatalysts.

This is further studied in the form of Tafel slope and electrochemical impedance spectroscopy analysis (EIS). The Tafel slope is one of the crucial factors associated with the OER reaction kinetics of the catalyst, suggesting that lowest Tafel slopes have faster reaction kinetics. It was estimated using the LSV curves shown in Figure 4a. Figure 4d shows the Tafel plot of the M_2_NiMnO_6_ (M = Eu, Gd, Tb) catalyst electrodes, and its values are presented in Table S2. As expected from the LSV results, the Tb_2_NiMnO_6_ exhibited the lowest Tafel slope of 38.76 mv dec^−1^ equated with Eu_2_NiMnO_6_, Gd_2_NiMnO_6_, and some of the double perovskites reported in the literature [34,35,36,37,38]. Therefore, the lowest Tafel slope of Tb_2_NiMnO_6_ suggests that the catalyst has abundant and faster reaction kinetics. Additionally, low Tafel values suggest the formation of the surface-adsorbed species well before the rate-determining steps, indicating the existence of a large number of active sites on the surface of the electrode.

**Figure 5:**
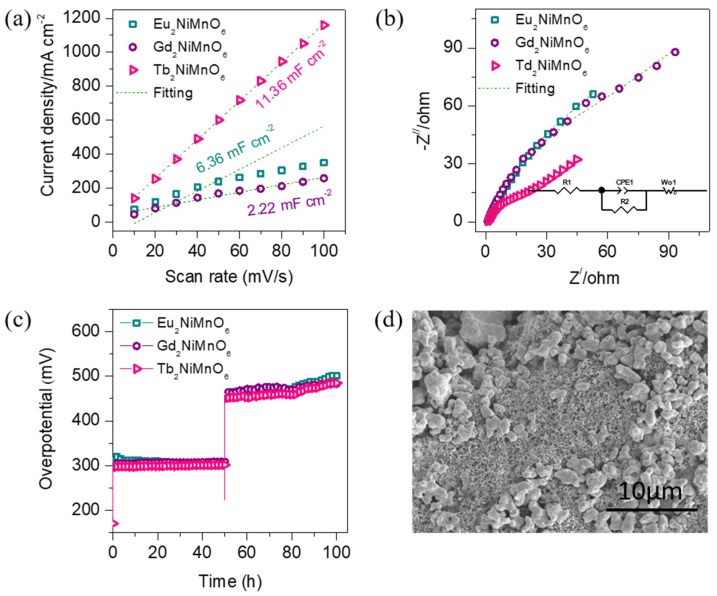
(**a**) Slope of the capacitive current (Δj) measured at a non-Faradaic voltage region V versus the scan rate, (**b**) Nyquist plots recorded at a 0-bias voltage and the inset equivalent circuit diagram used to fit the curves, (**c**) chronoamperometric stability curves measured at 10 and 100 mAcm^−2^ over 100 h, (**d**) SEM image of the double perovskite Tb_2_NiMnO_6_ catalyst electrode after 100 h of OER test depicting unchanged structural morphology.

The enhanced OER properties of the double perovskite catalyst electrodes were evaluated via ECSA and EIS analysis. ECSA was estimated by recording CV curves (Figure S3, Supporting Information) of all the catalyst electrodes at different scan rates of 10, 20, 30, 40, 50, 60, 70, 80 90, and 100 mVs^−1^ in non-faradaic regions. The current density obtained at a specific voltage of 0.15 V is plotted versus scan, which is shown in Figure 5a. The ECSA of the catalyst was calculated using Equation (1) [43].

ECSA = C_dl_/C_s_ (1)

where C_S_ is the specific capacitance in an alkaline medium (0.040 mF cm^−2^ for the KOH electrolyte) and C_dl_ is the specific capacitance of the double-layer region. C_dl_ is the slope of the curves in Figure 5a, which is found to be 11.36 mF cm^−1^ higher for the Tb_2_NiMnO_6_, whereas for the Eu_2_NiMnO_6_ and Gd_2_NiMnO_6_, it is 6.36 and 2.22 mF cm^−1^, respectively. Thus, the ECSA values presented in Table S1 (Supporting Information) indicate that the Tb_2_NiMnO_6_ catalyst has the highest ECSA of the 284 cm^2^, promoting OER properties. The intrinsic catalytic activity of the electrodes is determined by normalizing the LSV curves via ECSA values. Figure S4 (Supporting Information) shows the ECSA-normalized LSV curves of the double perovskite M_2_NiMnO_6_ (M = Eu, Gd, Tb) catalysts. Interestingly, upon ECSA normalization, it was observed that the OER properties of the best-performing Tb_2_NiMnO_6_ catalyst were poor compared with that of the Eu_2_NiMnO_6_ catalyst, suggesting a major contribution of the ECSA in the OER enhancement.

The electronic conductivity of the catalysts was also evaluated using EIS measurements for all the samples. Figure 5b shows the Nyquist plots of the OER catalysts, which are recorded at zero volt. All the Nyquist plots show a semicircle in the high-frequency region and a straight line in the low-frequency region, which is associated with the charge transfer resistance and diffusion coefficient of the electrode, respectively. All the curves were fitted using an equivalent circuit diagram (inset of Figure 5b) in the Z_view_ impedance fitting software and its parameters are presented in Table S3 (Supporting Information). As expected, the Tb_2_NiMnO_6_ catalyst had the lowest charge transfer resistance of 16.9 Ω compared with the other two catalysts, suggesting faster reaction kinetics and mass transport during the OER process.

We also investigated the long-term electrochemical stability of all the double perovskite catalysts in 1M KOH electrolyte using the chronopotentiometry technique. Figure 5c shows the chronopotentiometry curves (without *iR* correction) of the M_2_NiMnO_6_ (M = Eu, Gd, Tb) recorded at two different current densities of 10 and 100 mA cm^−1^ for 100 h. The rigorous evolution of the evolved gasses at the cathode and anode was observed during the stability test measurements. The best-performing catalyst Tb_2_NiMnO_6_ maintained the steady-state low overpotential without deviation at lower current rates, whereas there were slight fluctuations at high current rates. The fluctuations at high currents were due to the larger amount of gas generation and a bubbling effect at the electrode surface during the OER process. Overall, all the studied catalysts were stable over the range, suggesting excellent structural stability for alkaline electrolyzers. To assert the exceptional stability of the Tb_2_NiMnO_6_ in the alkaline electrolyte, the XRD pattern after the OER test was obtained. The X-ray diffraction peaks after the OER Figure S5 (Supporting Information) test are well matching with that of the before test seen in Figure 1c, except an additional peak at 35.76° suggesting the outstanding long-term stability of the electrode. Moreover, the additional peak seen at 35.76° is associated with the Fe_2_O_3_ phase due to oxidation of the stainless-steel substrate, which was obvious during the long-term OER test.

**Figure 6:**
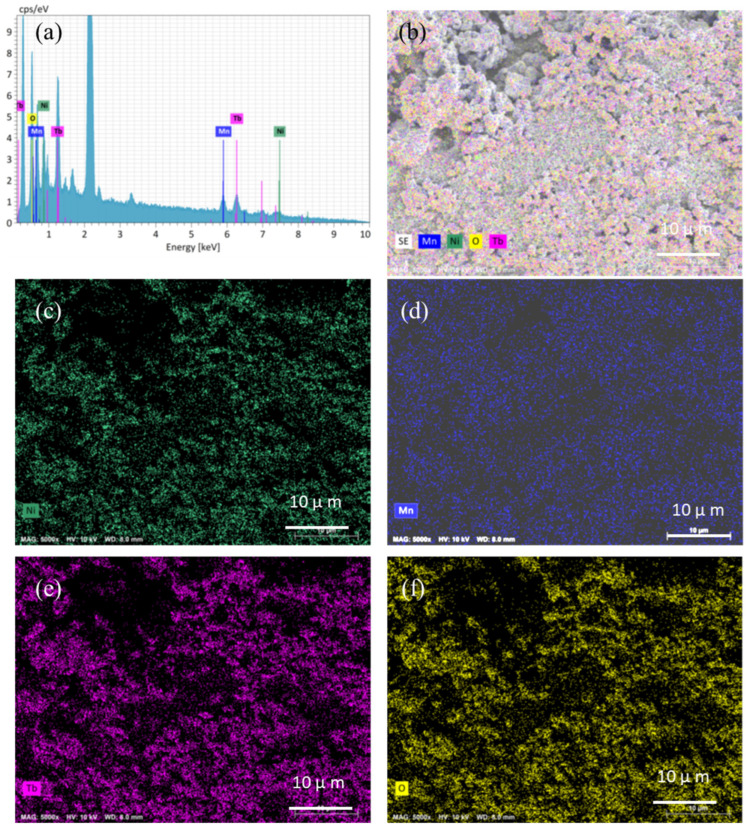
Elemental mapping analysis of double perovskite Tb_2_NiMnO_6_ catalyst electrode after a 100 h stability test in KOH electrolyte.

The surface morphologies after the OER test also provide useful information about the stability of the catalyst. The unchanged morphological image is shown in Figure 5d after the OER test and there is a slight difference in the shape of the LSV curve (Figure S6 Supporting Information) for Tb_2_NiMnO_6_, revealing the outstanding electrochemical stability of the electrode in the alkaline medium. Moreover, the EDAX and elemental mapping images after the OER test are shown in Figure 6. From the EDAX analysis (Figure 6a), it is seen that the Mn content of the catalyst slightly reduced, which was obvious during the catalysis process. This could be due to the structural changes, surface reconstruction to a catalytically active state, and dissolution of the electrode during the OER test. Figure 6b depicts the mixed elemental mapping image for Tb_2_NiMnO_6_ after the OER test. We also observed the existence and uniform distribution of the constituent elements such as Ni (green (Figure 6c), Mn (blue (Figure 6d), Tb (pink (Figure 6e), and O (yellow) (Figure 6f) in the catalyst after the OER test. This phenomenon is crucial for the enhancement of the catalytic activity and its mechanism.

## 4. Conclusions

In summary, we have utilized electrocatalysts based on lanthanide earth metal-doped double perovskite M_2_NiMnO_6_ (M = Eu, Gd, Tb) for the OER study in an alkaline electrolyte. It has been evidenced from the structural and electrochemical characterization that A-site doping with different rare-earth metals with different ionic radii can alter the surface oxidation or valence states of B_2_ in double perovskite A_2_B_1_B_2_O_6_-type perovskites, facilitating the electrochemical behavior of the materials. The catalyst with the configuration of Tb_2_NiMnO_6_ exhibited the lowest overpotential and Tafel slope of 288 mV (@10 mAcm^−2^) and 38.76 mVdec^−1^, respectively, which surpasses the many double perovskite catalysts reported in the literature. Moreover, these catalysts are extremely stable in the alkaline electrode for more than 100 h at different current rates while generating vigorous gasses. Thus, the strategy presented in this work could be one of the best strategies to fabricate stable and active catalyst materials.

## Data Availability

Data are contained within the article.

## Supplementary Materials

The following supporting information can be downloaded at: https://www.mdpi.com/article/10.3390/nano13233076/s1.

## References

[1] Zhao J.W., Shi Z.X., Li C.F., Ren Q., Li G.R. (2021). Regulation of Perovskite Surface Stability on the Electrocatalysis of Oxygen Evolution Reaction. ACS Mater. Lett..

[2] Wang P., Luo Y., Zhang G., Chen Z., Ranganathan H., Sun S., Shi Z. (2022). Interface Engineering of NixSy@MnOxHy Nanorods to Efficiently Enhance Overall-Water-Splitting Activity and Stability. Nano-Micro Lett..

[3] Serra J.M., Borrás-Morell J.F., García-Baños B., Balaguer M., Plaza-González P., Santos-Blasco J., Catalán-Martínez D., Navarrete L., Catalá-Civera J.M. (2020). Hydrogen production via microwave-induced water splitting at low temperature. Nat. Energy.

[4] Inamdar A.I., Chavan H.S., Hou B., Lee C.H., Lee S.U., Cha S., Kim H., Im H. (2020). A Robust Nonprecious CuFe Composite as a Highly Efficient Bifunctional Catalyst for Overall Electrochemical Water Splitting. Small.

[5] Zhang B., Qi Z., Wu Z., Lui Y.H., Kim T.H., Tang X., Zhou L., Huang W., Hu S. (2019). Defect-Rich 2D Material Networks for Advanced Oxygen Evolution Catalysts. ACS Energy Lett..

[6] Roy B., Sebok S.B., Scott E.M., Fiordaliso J.E., Sørensen A., Bodin D.B., Trimarco C.D., Damsgaard P.C.K., Vesborg O., Hansen I.E.L. (2018). Chorkendorff, Impact of nanoparticle size and lattice oxygen on water oxidation on NiFeOxHy. Nat. Catal..

[7] Chavan H.S., Lee C., Inamdar A.I., Han J., Park S., Cho S., Shreshta N.K., Lee S., Hou B., Im H. (2022). Designing and Tuning the Electronic Structure of Nickel–Vanadium Layered Double Hydroxides for Highly Efficient Oxygen Evolution Electrocatalysis. ACS Catal..

[8] Inamdar A.I., Chavan H.S., Seok J.H., Lee C.H., Shin G., Park S., Yeon S., Cho S., Park Y., Shrestha N.K. (2022). Optimal rule-of-thumb design of NiFeMo layered double hydroxide nanoflakes for highly efficient and durable overall water-splitting at large currents. J. Mater. Chem. A.

[9] Qu Y., Yang M., Chai J., Tang Z., Shao M., Kwok C.T., Yang M., Wang Z., Chua D., Wang S. (2017). Facile Synthesis of Vanadium-Doped Ni_3_S_2_ Nanowire Arrays as Active Electrocatalyst for Hydrogen Evolution Reaction. ACS Appl. Mater. Interfaces.

[10] Yu X.Y., Feng Y., Guan B.Y., Lou X.W., Paik U. (2016). Carbon coated porous nickel phosphides nanoplates for highly efficient oxygen evolution reaction. Energy Environ. Sci..

[11] Du J., Zou Z., Liu C., Xu C. (2018). Hierarchical Fe-doped Ni_3_Se_4_ ultrathin nanosheets as an efficient electrocatalyst for oxygen evolution reaction. Nanoscale.

[12] Wang X., Dai J., Zhou C., Guan D., Wu X., Zhou W., Shao Z. (2021). Engineering Charge Redistribution within Perovskite Oxides for Synergistically Enhanced Overall Water Splitting. ACS Mater. Lett..

[13] Zu M.Y., Wang C., Zhang L., Zheng L.R., Yang H.G. (2019). Reconstructing bimetallic carbide Mo6Ni6C for carbon interconnected MoNi alloys to boost oxygen evolution electrocatalysis. Mater. Horiz..

[14] Ma W., Wang M., Tan C., Wang J., Dai Y., Hu L., Lv X., Li Q., Dang J. (2023). Formulating a heterolytic cleavage process of water on Ni_3_N nanosheets through single transition metal doping for ultra-efficient alkaline hydrogen evolution. Inorg. Chem. Front..

[15] Hao J., Zhuang Z., Cao K., Gao G., Wang C., Lai F., Lu S., Ma P., Dong W., Liu T. (2022). Unraveling the electronegativity-dominated intermediate adsorption on high-entropy alloy electrocatalysts. Nat. Commun..

[16] Shinde K.P., Lee E.J., Manawan M., Lee A., Park S.-Y., Jo Y., Ku K., Kim J.M., Park J.S. (2021). Structural, magnetic, and magnetocaloric properties of R_2_NiMnO_6_ (R = Eu, Gd, Tb). Sci. Rep..

[17] Meng Z., Xu J., Yu P., Hu X., Wu Y., Zhang Q., Li Y., Qiao L., Zeng Y., Tian H. (2020). Double perovskite La_2_CoMnO_6_ hollow spheres prepared by template impregnation for high-performance supercapacitors. Chem. Eng. J..

[18] Kim M.K., Moon J.Y., Choi H.Y., Oh S.H., Lee N., Choi Y.J. (2015). Effects of different annealing atmospheres on magnetic properties in La_2_CoMnO_6_ single crystals. Curr. Appl. Phys..

[19] Yin W.J., Weng B.C., Ge J., Sun Q.D., Li Z.Z., Yan Y.F. (2019). Oxide perovskites, double perovskites and derivatives for electrocatalysis, photocatalysis, and photovoltaics. Energy Environ. Sci..

[20] Alam M., Karmakar K., Pal M., Mandal K. (2016). Electrochemical supercapacitor based on double perovskite Y_2_NiMnO_6_ nanowires. RSC Adv..

[21] Singh J., Kumar A. (2019). Facile wet chemical synthesis and electrochemical behavior of La_2_FeCoO_6_ nano-crystallites. Mater. Sci. Semicond. Process..

[22] Kumar A., Kumar A., Kumar A. (2020). Energy storage properties of double perovskites Gd_2_NiMnO_6_ for electrochemical supercapacitor application. Solid State Sci..

[23] Liu Y., Zhang J., Li Y., Qian Q., Li Z., Zhu Y., Zhang G. (2020). Manipulating dehydrogenation kinetics through dual-doping Co_3_N electrode enables highly efficient hydrazine oxidation assisting self-powered H_2_ production. Nat. Commun..

[24] Zhuang P., Sun Y., Dong P., Smith W., Sun Z., Ge Y., Pei Y., Cao Z., Ajayan P.M., Shen J. (2019). Revisiting the Role of Active Sites for Hydrogen Evolution Reaction through Precise Defect Adjusting. Adv. Funct. Mater..

[25] Sheikh S., Ghosh D., Dutta A., Bhattacharyya S., Sinha T.P. (2017). Lead free double perovskite oxides Ln_2_NiMnO_6_(Ln=La, Eu, Dy, Lu), a new promising material for photovoltaic application. Mater. Sci. Eng. B.

[26] Klimkowicz A., Świerczek K., Zheng K., Baranowska M., Takasaki A., Dabrowski B. (2014). Evaluation of BaY_1−x_Pr_x_Mn_2_O_5+δ_ oxides for oxygen storage technology. Solid State Ion..

[27] Zankowski S.P., Hoecke L.V., Mattelaer F., De Raedt M., Richard O., Detavernier C., Vereecken P.M. (2019). Redox layer deposition of thin films of MnO2 on nanostructured substrates from aqueous solutions. Chem. Mater..

[28] Masud M.G., Sakata H., Biswal A.K., Vishwakarma P.N., Chaudhuri B.K. (2015). Structural, ac conductivity scaling and magnetodielectric behaviour of a partially disordered insulating ferromagnetic double perovskite Eu_2_NiMnO_6_. J. Phys. D Appl. Phys..

[29] Wang T., Wu H.-Y., Xing R., Sun Y.-B., Xv B., Zhao J.-J. (2019). Physical Properties of Ca-Doped Double Perovskite La_2_NiMnO_6_. J. Low Temp. Phys..

[30] Zhang W., Shen H., Yin M., Lu L., Xu B., Li D. (2022). Heterostructure Silicon Solar Cells with Enhanced Power Conversion Efficiency Based on Si_x_/Ni^3+^ Self-Doped NiOx Passivating Contact. ACS Omega.

[31] Wang M., Chen K., Liu J., He Q., Li G., Li F. (2018). Efficiently Enhancing Electrocatalytic Activity of α-MnO_2_ Nanorods/N-Doped Ketjenblack Carbon for Oxygen Reduction Reaction and Oxygen Evolution Reaction Using Facile Regulated Hydrothermal Treatment. Catalysts.

[32] Gupta P., Mahapatra P.K., Choudhary R.N.P. (2020). TbFeO_3_Ceramic: An Exciting Colossal Dielectric with Ferroelectric Properties. Phys. Status Solidi B.

[33] Yi K., Tang Q., Wu Z., Zhu X. (2022). Unraveling the Structural, Dielectric, Magnetic, and Optical Characteristics of Nanostructured La_2_NiMnO_6_ Double Perovskites. Nanomaterials.

[34] Diaz-Morales O., Raaijman S., Kortlever R., Kooyman P.J., Wezendonk T., Gascon J., Fu W.T., Koper M.T.M. (2016). Iridium-based double perovskites for efficient water oxidation in acid media. Nat. Commun..

[35] Banerjee A., Awasthi M.K., Maji P., Pal M., Aziz S.T., Lahiri G.K., Dutta A. (2023). Double Perovskite Oxides Bringing a Revelation in Oxygen Evolution Reaction Electrocatalyst Design. ChemElectroChem.

[36] Miao X., Wu L., Lin Y., Yuan X., Zhao J., Yan W., Zhou S., Shi L. (2019). The role of oxygen vacancies in water oxidation for perovskite cobalt oxide electrocatalysts: Are more better?. Chem. Commun..

[37] Qu M., Ding X., Shen Z., Cui M., Oropeza F.E., Gorni G., de la Peña O’Shea V.A., Li W., Qi D.-C., Zhang K.H.L. (2021). Tailoring the Electronic Structures of the La_2_NiMnO_6_ Double Perovskite as Efficient Bifunctional Oxygen Electrocatalysis. Chem. Mater..

[38] Wang J., Gao Y., Chen D., Liu J., Zhang Z., Shao Z., Ciucci F. (2018). Water Splitting with an Enhanced Bifunctional Double Perovskite. ACS Catal..

[39] Zhu J., Guđmundsdóttir J.B., Strandbakke R., Both K.G., Aarholt T., Carvalho P.A., Sørby M.H., Jensen I.J.T., Guzik M.N., Norby T. (2021). Double Perovskite Cobaltites Integrated in a Monolithic and Noble Metal-Free Photoelectrochemical Device for Efficient Water Splitting. ACS Appl. Mater. Interfaces.

[40] Sun H., Chen G., Sunarso J., Dai J., Zhou W., Shao Z. (2018). Molybdenum and Niobium Codoped B-Site-Ordered Double Perovskite Catalyst for Efficient Oxygen Evolution Reaction. ACS Appl. Mater. Interfaces.

[41] Guo X., Zheng X., Hu X., Zhao Q., Li L., Yu P., Jing C., Zhang Y., Huang G., Jiang B. (2021). Electrostatic adsorbing graphene quantum dot into nickel–based layered double hydroxides: Electron absorption/donor effects enhanced oxygen electrocatalytic activity. Nano Energy.

[42] Higareda A., Hernández-Arellano D.L., Ordoñez L.C., Barbosa R., Alonso-Vante N. (2023). Advanced Electrocatalysts for the Oxygen Evolution Reaction: From Single- to Multielement Materials. Catalysts.

[43] Inamdar A.I., Chavan H.S., Pawar S.M., Kim H., Im H. (2020). NiFeCo oxide as an efficient and sustainable catalyst for the oxygen evolution reaction. Int. J. Energy Res..

