# Supplementary material for: Electrochemical Investigations of Double Perovskite M2NiMnO6 (Where M = Eu, Gd, Tb) for High-Performance Oxygen Evolution Reaction
Source: Nanomaterials (Basel). 2023 Dec 4;13(23):3076. doi: 10.3390/nano13233076 (PMC10707741; doi:10.3390/nano13233076)
Supplement: Supplementary file 1 [file nanomaterials-13-03076-s001.zip › nanomaterials-2751593-supplementary.pdf]

Supporting Information

Electrochemical Investigations of Double Perovskite M_2NiMnO_6 (Where M = Eu, Gd, Tb) for High-Performance Oxygen Evolution Reaction

Kiran P. Shinde [1], Harish S. Chavan [2], Amol S. Salunke [3], Jeongseok Oh [1], Abu Talha Aqueel Ahmed [3], Nabeen K. Shrestha [3], Hyunsik Im [3], Joonsik Park [1] and Akbar I. Inamdar [3,*]

[1] Department of Materials Science and Engineering, Hanbat National University, Daejeon 34158, Republic of Korea
[2] Department of Chemistry and Chemical Engineering, Inha University, 100 Inha-ro, Michuhol-gu, Incheon 22212, Republic of Korea
[3] Division of Physics and Semiconductor Science, Dongguk University, Seoul 04620, Republic of Korea
[*] Correspondence: akbarphysics2002@gmail.com

(a)

(b)

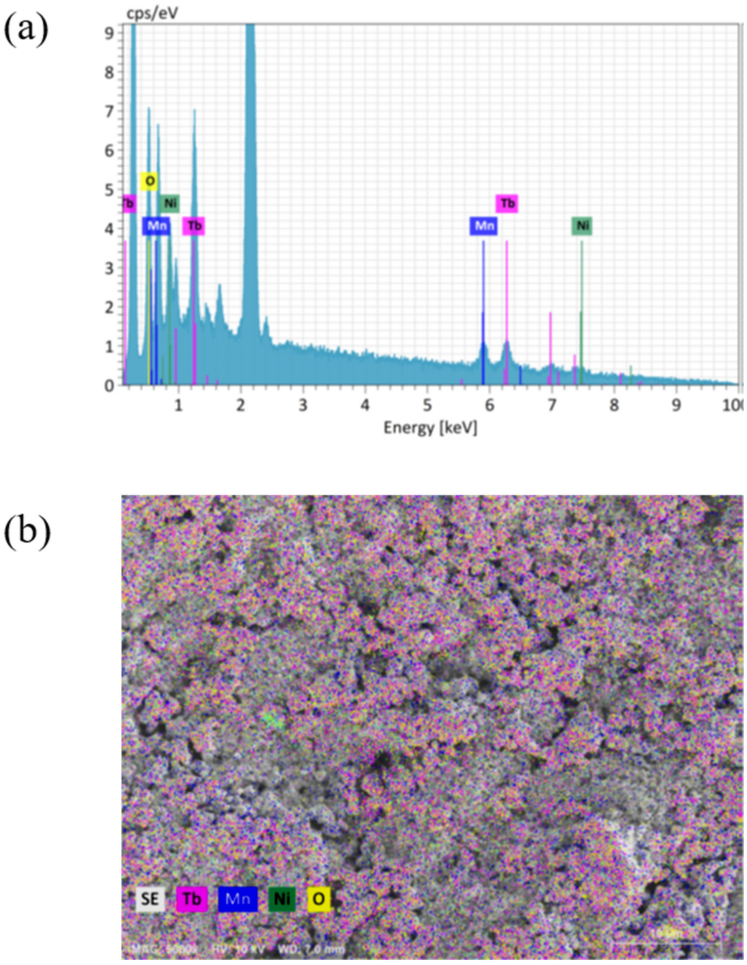

Figure S1. (a) Energy dispersive X-ray analysis (EDAX) spectra of the double perovskite Tb_2NiMnO_6 catalyst electrode, indicating the presence of all the constituent elements in the catalyst. **(b)** Mixed elemental mapping image of the double perovskite Tb_2NiMnO_6 catalyst electrode.

Table S1. Structural parameters for M_2NiMnO_6 (where M= Eu, Gd, Tb) from the Rietveld refinement

Cell parameters	**Eu_2NiMnO_6**	**Gd_2NiMnO_6**	**Tb_2NiMnO_6**
Space group	$P2_1/n$	$P2_1/n$	$P2_1/n$
Atomic number M2	63	64	65
Crystal density g/cm^3	7.65	7.86	7.98
Cell volume (Å^3)	222.9	221.6	219.6

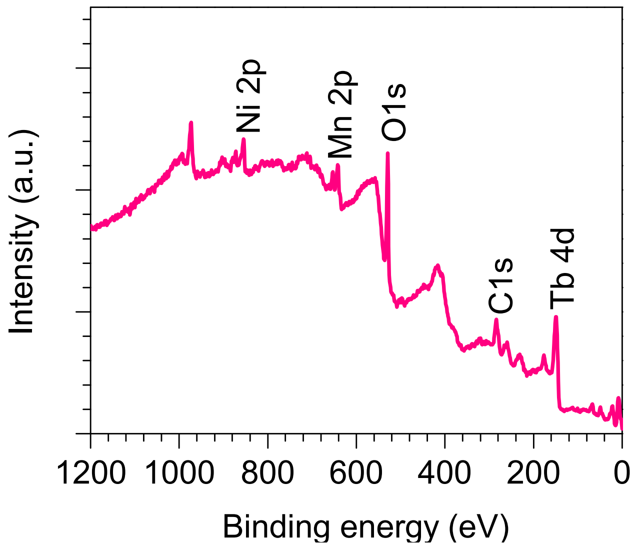

Figure S2. XPS survey spectra of the double perovskite Tb_2NiMnO_6 catalyst electrode depicting Ni. Mn. Tb and O in the sample.

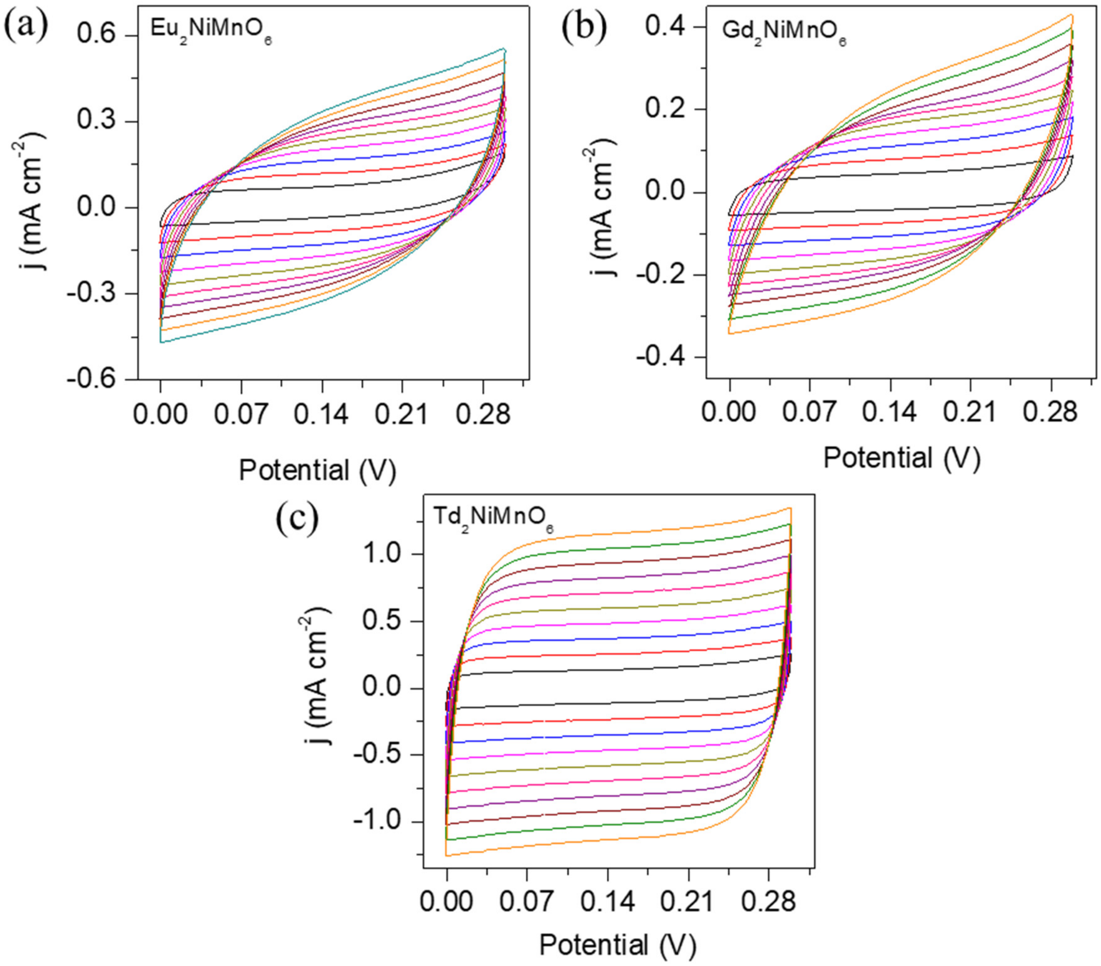

Figure S3. Cyclic voltammetry curves recorded at different scan rates of 10, 20, 30, 40, 50, 60, 70, 80, 90, and 100 mVs^{-1} in non-faradaic voltage region for **(a)** Eu_2NiMnO_6, **(b)** Gd_2NiMnO_6, **(c)** Tb_2NiMnO_6.

Table S2. Electrochemical water splitting parameters for OER analysis for the double perovskite M_2NiMnO_6 (M= Eu, Gd, Tb) compounds.

Sample name	Overpotential (mV) @ 10 mAcm^{-2}	Tafel slop (mV dec^{-1})	ECSA (cm^{2})
**Eu**	334	52.3	159
**Gd**	292	45.11	55.5
**Td**	288	38.76	284

Table S3. Parameters determined from fitting of the electrochemical impedance spectroscopy (EIS) curves using equivalent circuit diagram.

Sample name	EIS @ 0V		EIS @ 0.4 V	
	Rs (Ω)	Rct (Ω)	Rs (Ω)	Rct (Ω)
**Eu**	**0.987**	**134.7**	**0.826**	**1.023**
**Gd**	**0.833**	**69.3**	**0.230**	**0.830**
**Td**	**0.984**	**16.9**	**1.034**	**0.698**

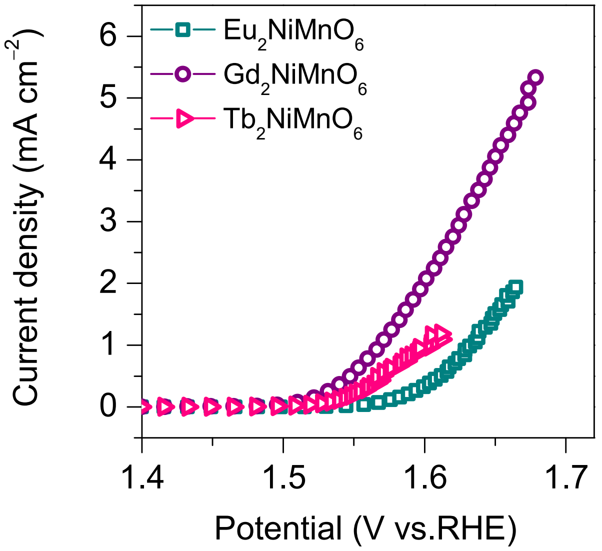

Figure S4. The ECSA normalized LSV curves of the double perovskite M_2NiMnO_6 catalyst electrodes to know the intrinsic catalytic activity of the electrode.

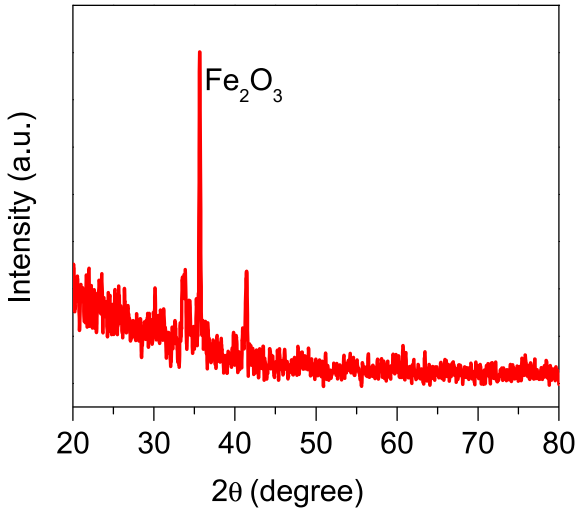

Figure S5. X-ray diffraction pattern of the Tb_2NiMnO_6 after long term OER test in alkaline electrolyte.

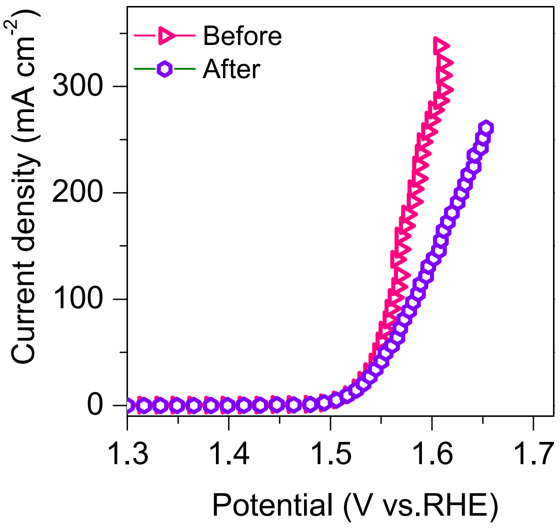

Figure S6. The *iR*-corrected LSV curves of double perovskite Tb_2NiMnO_6 catalyst electrode before and after stability test of 100 hours confirm its excellent electrochemical stability.
